# p38MAPK/SGK1 signaling regulates macrophage polarization in experimental autoimmune encephalomyelitis

**DOI:** 10.18632/aging.101786

**Published:** 2019-02-04

**Authors:** Bo Li, Tian-Bi Tan, Liang Wang, Xiao-Yun Zhao, Guo-Jun Tan

**Affiliations:** 1 Department of Neurology, Bethune International Peace Hospital, Shijiazhuang 050000, China; 2 Dynacare, Ottawa, Ontario, K1L8H2, Canada; 3 Department of Neurology, the Second Hospital of Hebei Medical University, Shijiazhuang 050000, China; 4 Key Laboratory of Hebei Neurology, Shijiazhuang 050000, China; 5 Laboratory Medicine Center of Qilu Hospital of Shandong University (Qingdao), Qingdao 266035, China

**Keywords:** multiple sclerosis (MS), experimental autoimmune encephalomyelitis (EAE), macrophage polarization, p38MAPK/SGK1 signaling

## Abstract

Multiple sclerosis (MS) is characterized with multifocal demyelination resulting from activation and infiltration of inflammatory cells into the central nerve system. Recent reports suggest that p38 mitogen-activated protein kinase (MAPK) / serum- and glucocorticoid-inducible protein kinase 1 (SGK1) signaling pathway contributes to the pathology of MS through regulation of immunity. However, the role of this signaling pathway in MS-related macrophage activation and polarization has not been studied. Here, we used an experimental autoimmune encephalomyelitis (EAE) model for MS to study the role of p38MAPK/SGK1 signaling in the macrophage polarization and its effects on the development and severity of EAE. Here, we found that p38MAPK/SGK1 signaling is required for IL4-induced M2 macrophage polarization in vitro. Chitin-induced M2 macrophage polarization reduces the severity of EAE in mice. Generation of an adeno-associated virus (AAV) carrying sh-p38 or sh-SGK1 under the control of a CD68 promoter successfully knockdown p38 or SGK1 levels in vitro and in vivo. Treatment with AAV-sh-p38 or AAV-sh-SGK1 abolished the effects of Chitin on macrophage polarization and the severity of EAE. Thus, our data suggest that p38MAPK/SGK1 signaling induces M2 macrophage polarization, which reduces the severity of EAE, a model for MS.

## Introduction

Multiple sclerosis (MS) is a severe disease of the central nervous system (CNS), characterized with a specific neurological disorder called multifocal demyelination resulting from activation and infiltration of inflammatory cells into the CNS [1]. The most important inflammatory cells that participate into the initiation, progression and development of MS are antigen-specific T cells (Th1 and Th17 cells appear to be the critical ones) and macrophages [2]. Although the T cells have been extensively studies for their role and functions in the pathogenesis of MS, the exact role and functions of macrophages remain poorly understood.

In the initial phase of MS, peripheral macrophages are recruited to the CNS, where they participate in the initiation, progression and development of disease, through their interaction with other inflammatory cells and residential microglia [3]. During the early phase, microglia/macrophages are predominantly activated to differentiate into classically activated macrophages (also called M1 macrophages) that release pro-inflammatory cytokines to enhance their damage to CNS tissue [4]. During the later phase, microglia/macrophages in the inflamed CNS are extensively polarized to alternatively activated macrophage phenotype (also called M2 macrophages) that release anti-inflammatory and trophic cytokines to favor inflammation repression and tissue recovery [4]. The dynamic change in the balance between M1 and M2 macrophages in the CNS determine the progression, severity and outcome of the disease [4]. While M1 macrophage polarization is induced by pro-inflammatory cytokine interferon-gamma (IFN-γ), M2 macrophage polarization is primarily induced by interleukin (IL)-4 and IL-13 [5].

The p38 mitogen-activated protein kinase (MAPK) / serum- and glucocorticoid-inducible protein kinase 1 (SGK1) signaling pathway mediates a number of important inflammatory reactions in response to a variety of stimuli [6]. Recent studies have identified this signaling pathway as a key player in the pathology of MS through regulating crucial immunopathogenic events. For example, in MS, p38MAPK/SGK1 signaling regulates cell death and survival, and contribute to cytokine production and secretion [7]. However, the role of this signaling pathway in MS-related macrophage activation and polarization has not been studied and was addressed in the current study.

The classical and most commonly applied animal model for MS is experimental autoimmune encephalomyelitis (EAE) in mice [8]. EAE exhibits most properties of MS, including demyelination, damage of axons, neurological dysfunction and degradation, and the pattern and pathology of the immune cell infiltration in the CNS [8]. Here, we used an EAE mouse model to study the role of p38MAPK/SGK1 signaling in the macrophage polarization and its effects on the development and severity of EAE.

## RESULTS

### IL-4 induces M2 macrophage polarization in vitro

**Figure 1:**
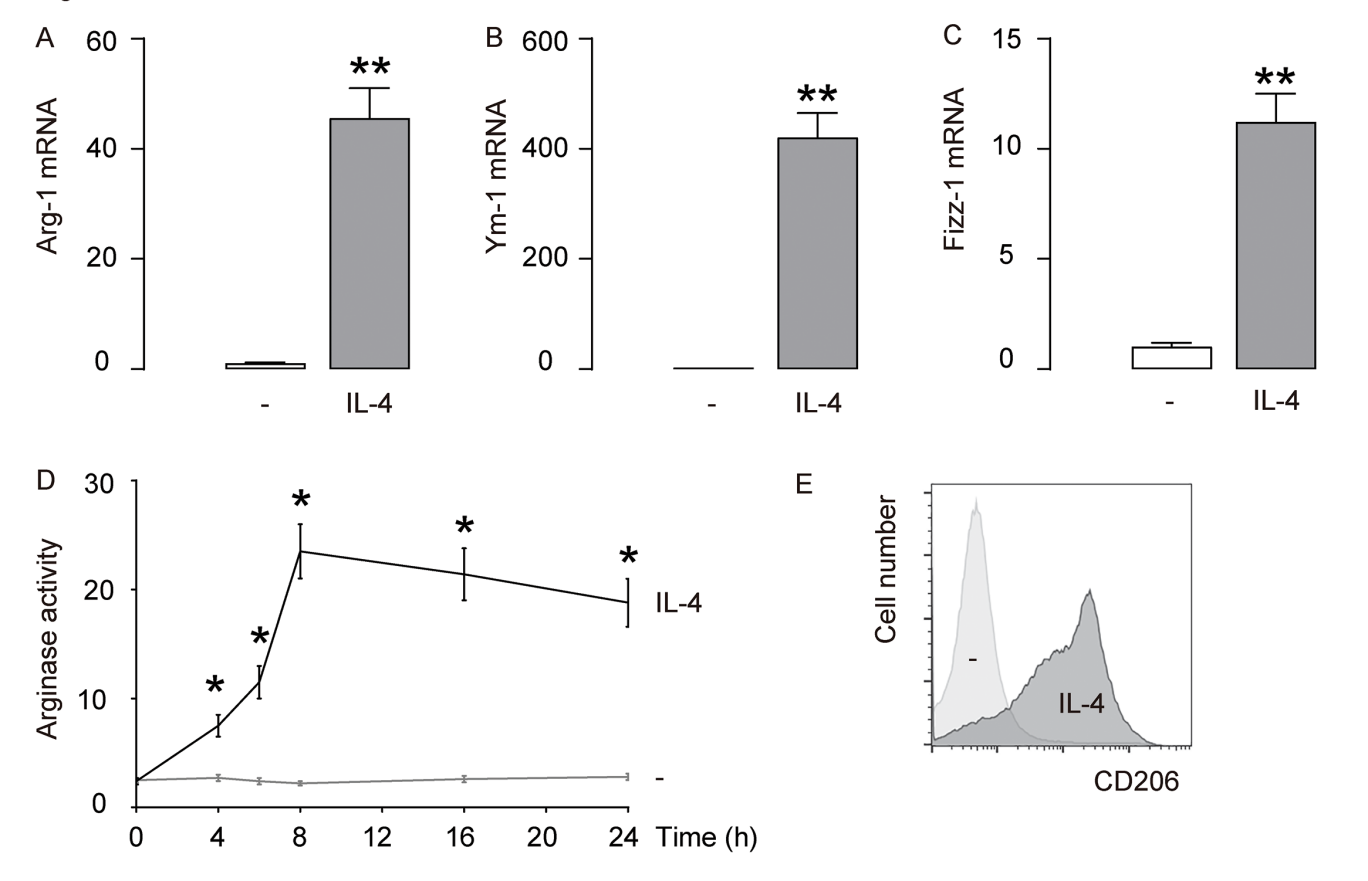
**IL-4 induces M2 macrophage polarization *in vitro.*** IL-4 was used to induce M2 macrophages from cultured macrophages derived from bone marrow in culture. (**A-C**) mRNA levels of Arginase 1 (Arg-1; **A**), Ym-1 (**B**) and Fizz-1 (**C**) by RT-qPCR. (**D**) Arginase assay. (**E**) Flow cytometry for CD206 in CTL (-) and IL-4-treated macrophages. **p<0.01. *p<0.05. N=5.

First, IL-4 was used to induce M2 macrophages from cultured macrophages derived from bone marrow in culture. To confirm the M2 macrophage polarization by IL-4, we examined the mRNA levels of Arginase 1 (Arg-1), Ym-1 and Fizz-1, three M2 macrophage markers. We found that IL-4 induced about 40 times’ increase in Arg-1 mRNA (Figure 1A), about 400 times’ increase in Ym-1 mRNA (Figure 1B), and about 10 times’ increase in Fizz-1 mRNA (Figure 1C). A typical characteristic of M2 macrophages is their potential role in converting L-arginine to ornithine and urea through the action of Arg-1. Thus, arginase activity was determined using a urea-based assay, showing significant increases in IL-4-treated macrophages (Figure 1D). CD206 is a surface marker exclusively expressed by M2 macrophages. We found that CD206 was induced in macrophages exposed to IL-4 by flow cytometry (Figure 1E). Together, these data suggest that IL-4-induces M2 macrophage polarization in vitro.

### p38MAPK/SGK1 signaling is required for IL-4-induced M2 macrophage polarization

**Figure 2:**
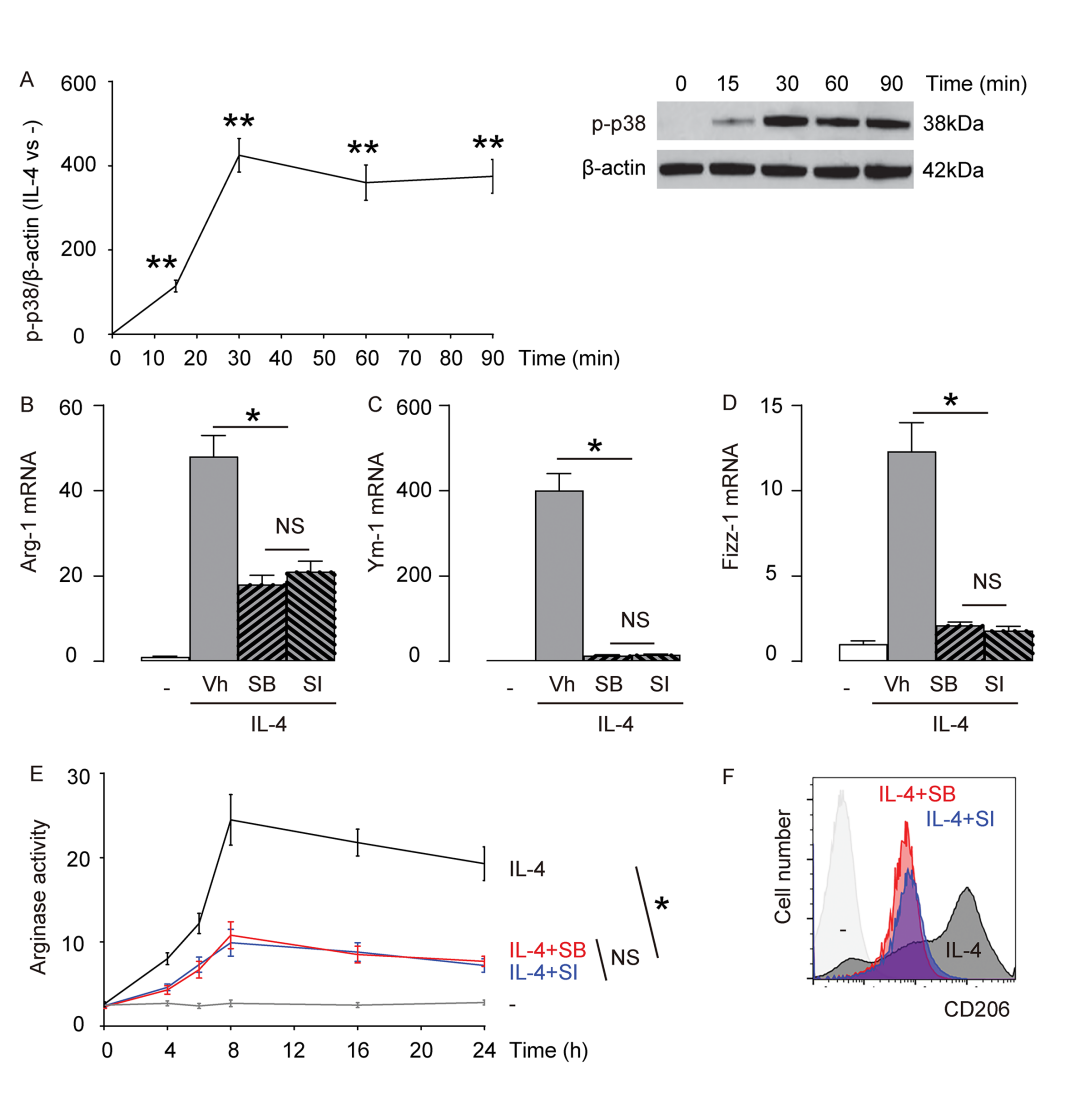
**p38MAPK/SGK1 signaling is required for IL-4-induced M2 macrophage polarization.** (**A**) The levels of phosphorylation of p38 (p-p38), an active form of p38, were examined in macrophages with time after exposure to IL-4 by Western blotting. (**B-F**) Bone marrow derived macrophages were pretreated with vehicle (Vh; DMSO), or a specific inhibitor of p38MAPK, SB203580 (SB), or a specific inhibitor of SGK1, SI113 (SI), previous to IL-4 stimulation. (**B-D**) mRNA levels of Arg-1 (**B**), Ym-1 (**C**) and Fizz-1 (**D**) by RT-qPCR. (**E**) Arginase assay. (**F**) Flow cytometry for CD206 in Vh, SB or SI-treated macrophages that were exposed to IL-4. **p<0.01. *p<0.05. NS: non-significant. N=5.

Previous reports have suggested that p38MAPK/SGK1 is one of the signaling pathways downstream of IL-4 stimulation [9–14]. Here, we examined the levels of phosphorylation of p38 (p-p38), an active form of p38, in macrophages with time after exposure to IL-4. We found that p-p38 was detected as early as 15 minutes after macrophages were exposed to IL-4, and the activation seemed to sustain at least for 90 minutes (Figure 2A). Next, we aimed to find out whether p38MAPK/SGK1 signaling may be required for IL-4-induced M2 macrophage polarization. Thus, bone marrow derived macrophages were pretreated with vehicle (Vh; DMSO), or a specific inhibitor of p38MAPK, SB203580 (SB), or a specific inhibitor of SGK1, SI113 (SI), previous to IL-4 stimulation. First, we examined the mRNA levels of Arg-1, Ym-1 and Fizz-1. We found that either SB, or SI significantly and similarly attenuated the IL-4-induced augmentation of Arg-1 mRNA (Figure 2B), Ym-1 mRNA (Figure 2C), and Fizz-1 mRNA (Figure 2D) in macrophages. Moreover, IL-4-induced increases in arginase activity in macrophages was also significantly and similarly attenuated by either SB, or SI (Figure 2E). Finally, we found that IL-4-induced expression of M2 surface marker, CD206, in macrophages was also significantly and similarly attenuated by either SB, or SI (Figure 2F). Together, these data suggest that p38MAPK/SGK1 signaling is required for IL-4-induced M2 macrophage polarization.

### Chitin-induced M2 macrophage polarization reduces the severity of EAE

**Figure 3:**
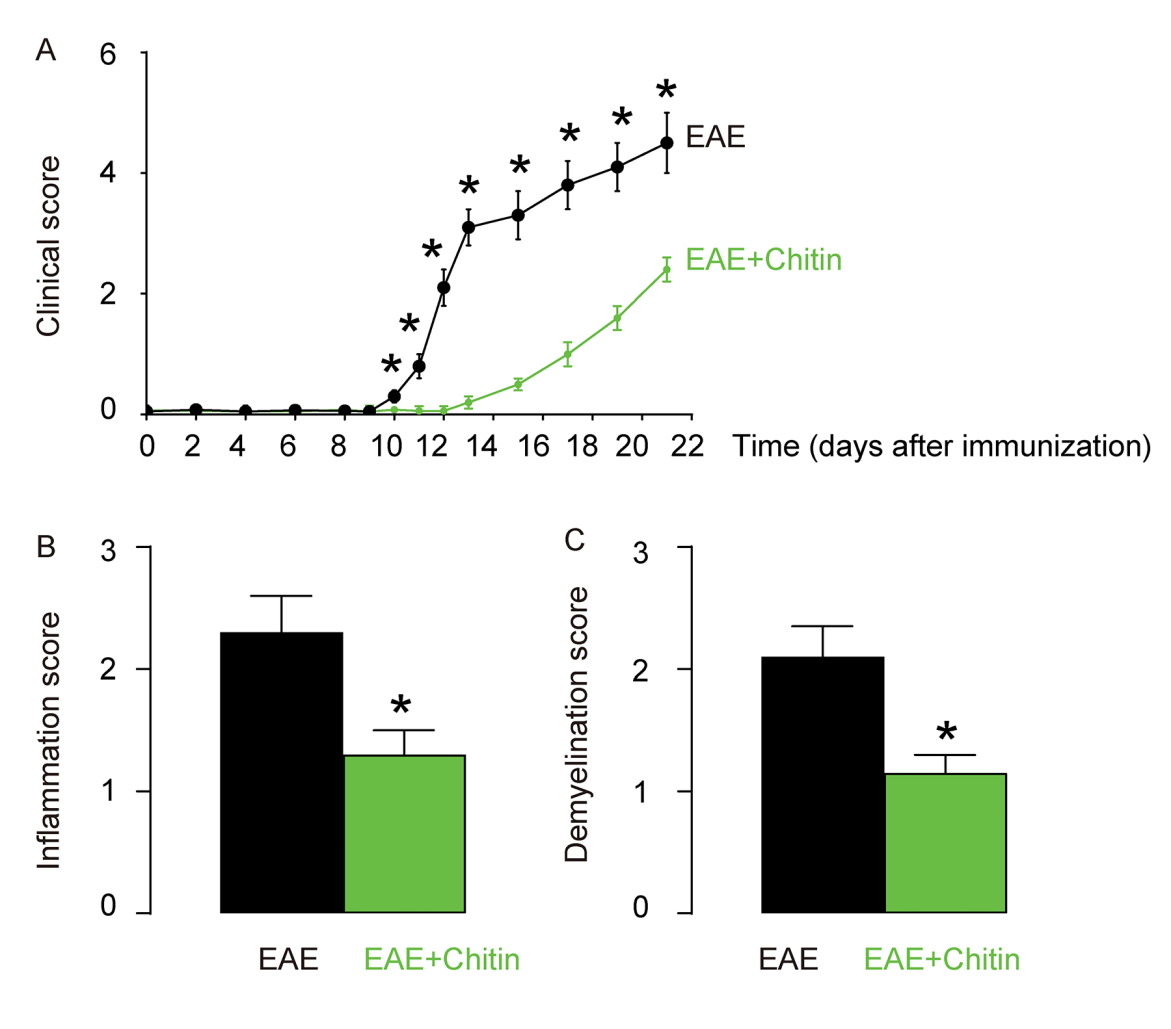
**Chitin-induced M2 macrophage polarization reduces the severity of EAE.** C57BL/6 mice were immunized with MOG_35-55_ in CFA to induce EAE. Some of the MOG_35-55_-treated mice were randomly selected to receive intraspinal injection of Chitin. The other MOG_35-55_-treated mice received intraspinal injection of same amount of DMSO as controls. The development and severity of clinical signs in the two groups of mice (EAE or EAE+Chitin) were monitored longitudinally till day 21 after immunization, when the mice were sacrificed to evaluate the pathological changes in the spinal cord. (**A**) The clinical score. (**B**) The inflammation score. (**C**) The demyelination score. *p<0.05. N=10.

Although inflammation and demyelination hallmark the pathology of EAE or MS, it is not clear whether macrophage polarization may play a role in the disease initiation, progression and severity. Administration of Chitin has been shown to induce IL-4-dependent recruitment and polarization of M2 macrophages [15,16]. Here, C57BL/6 mice were immunized with MOG_35-55_ in CFA to induce EAE. Some of the MOG_35-55_-treated mice were randomly selected to receive intraspinal injection of Chitin. The other MOG_35-55_-treated mice received intraspinal injection of same amount of DMSO as controls. The development and severity of clinical signs in the two groups of mice (EAE or EAE+Chitin) were monitored longitudinally till day 21 after immunization, when the mice were sacrificed to evaluate the pathological changes in the spinal cord. We found that Chitin administration significantly increased the M2 vs M1 macrophage ratio in the mouse brain by 16.6±1.8 folds, resulting in reduced the clinical score (Figure 3A), inflammation score (Figure 3B) and demyelination score (Figure 3C), suggesting that Chitin-induced M2 macrophage polarization reduces the severity of EAE.

### Generation of an adeno-associated virus (AAV) carrying sh-p38 or sh-SGK1 under the control of a CD68 promoter

**Figure 4:**
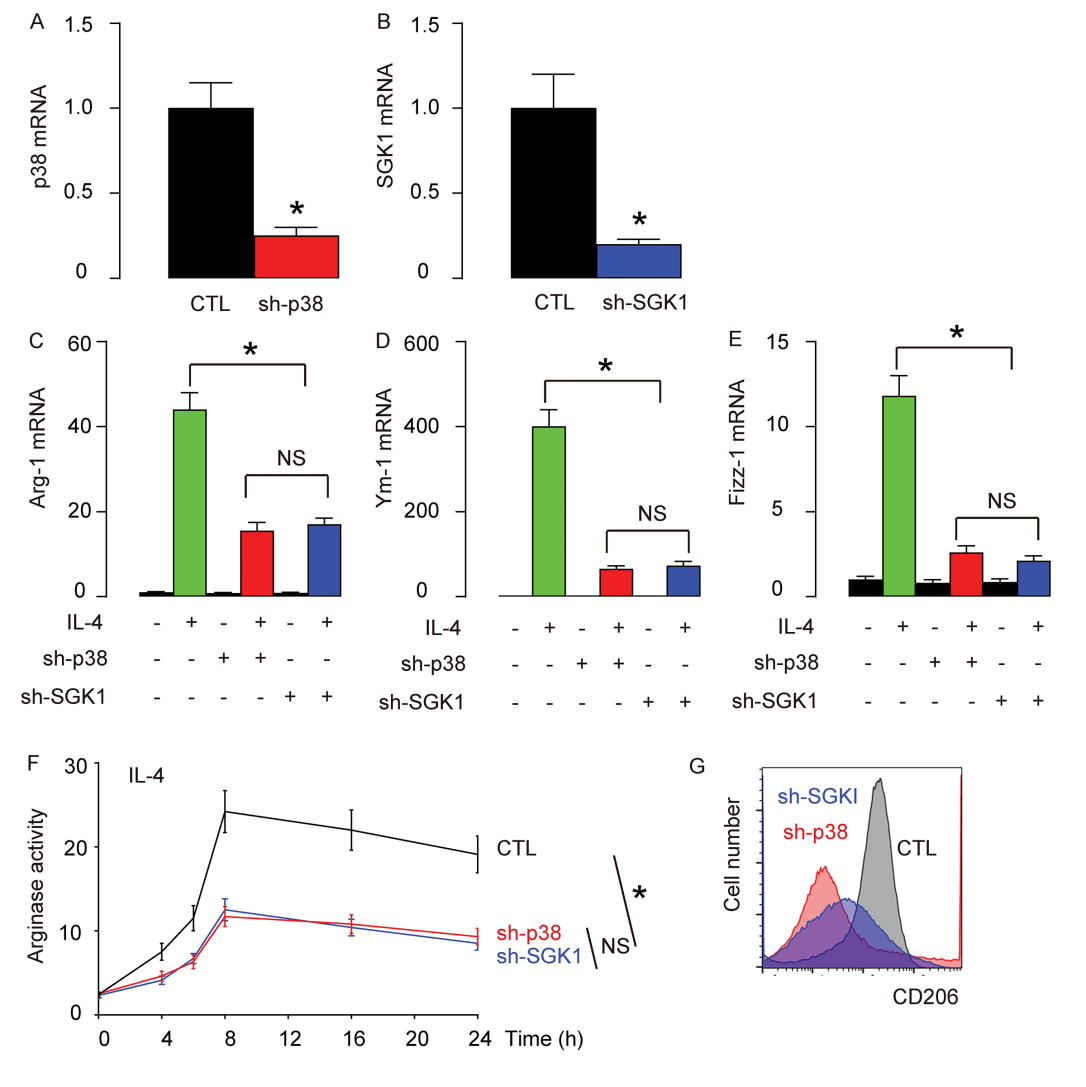
**Generation of an adeno-associated virus (AAV) carrying sh-p38 or sh-SGK1 under the control of a CD68 promoter.** An adeno-associated virus (AAV) carrying sh-p38 or sh-SGK1 under the control of a CD68 promoter (AAV-sh-p38, AAV-sh-SGK1) was generated to specifically knockdown p38 or SGK1 exclusively in macrophages. (**A-B**) RT-qPCR for p38 (A) or SGK1 (B) levels in AAV-sh-p38 or AAV-sh-SGK1-treated macrophages in vitro. (**C-E**) mRNA levels of Arg-1 (**C**), Ym-1 (**D**) and Fizz-1 (**E**) by RT-qPCR. (**F**) Arginase assay. (**G**) Flow cytometry for CD206 in control (CTL), sh-p38, or sh-SGK1-treated macrophages that were exposed to IL-4. *p<0.05. NS: non-significant. N=5.

In order to examine whether p38MAPK/SGK1 signaling may be required for the M2 macrophage polarization-induced reduction in the severity of EAE, we generated an adeno-associated virus (AAV) carrying sh-p38 or sh-SGK1 under the control of a CD68 promoter (AAV-sh-p38, AAV-sh-SGK1) to specifically knockdown p38 or SGK1 exclusively in macrophages. First, the reduction of p38 (Figure 4A) or SGK1 (Figure 4B) levels in AAV-sh-p38 or AAV-sh-SGK1-treated macrophages were confirmed in vitro. We found that depletion of either p38 or SGK1 significantly and similarly reduced the IL-4-induced activation of Arg-1 (Figure 4C), Ym-1 (Figure 4D) and Fizz-1 (Figure 4E) by RT-qPCR, significantly and similarly reduced the IL-4-induced increases in arginase activity (Figure 4F), and reduced IL-4-induced expression of CD206 (Figure 4G). Together, these data suggest that depletion of either p38 or SGK1 may reduce IL-4-induced M2 macrophage polarization in vitro.

### Treatment with AAV-sh-p38 or AAV-sh-SGK1 attenuates the effects of Chitin on the severity of EAE

**Figure 5:**
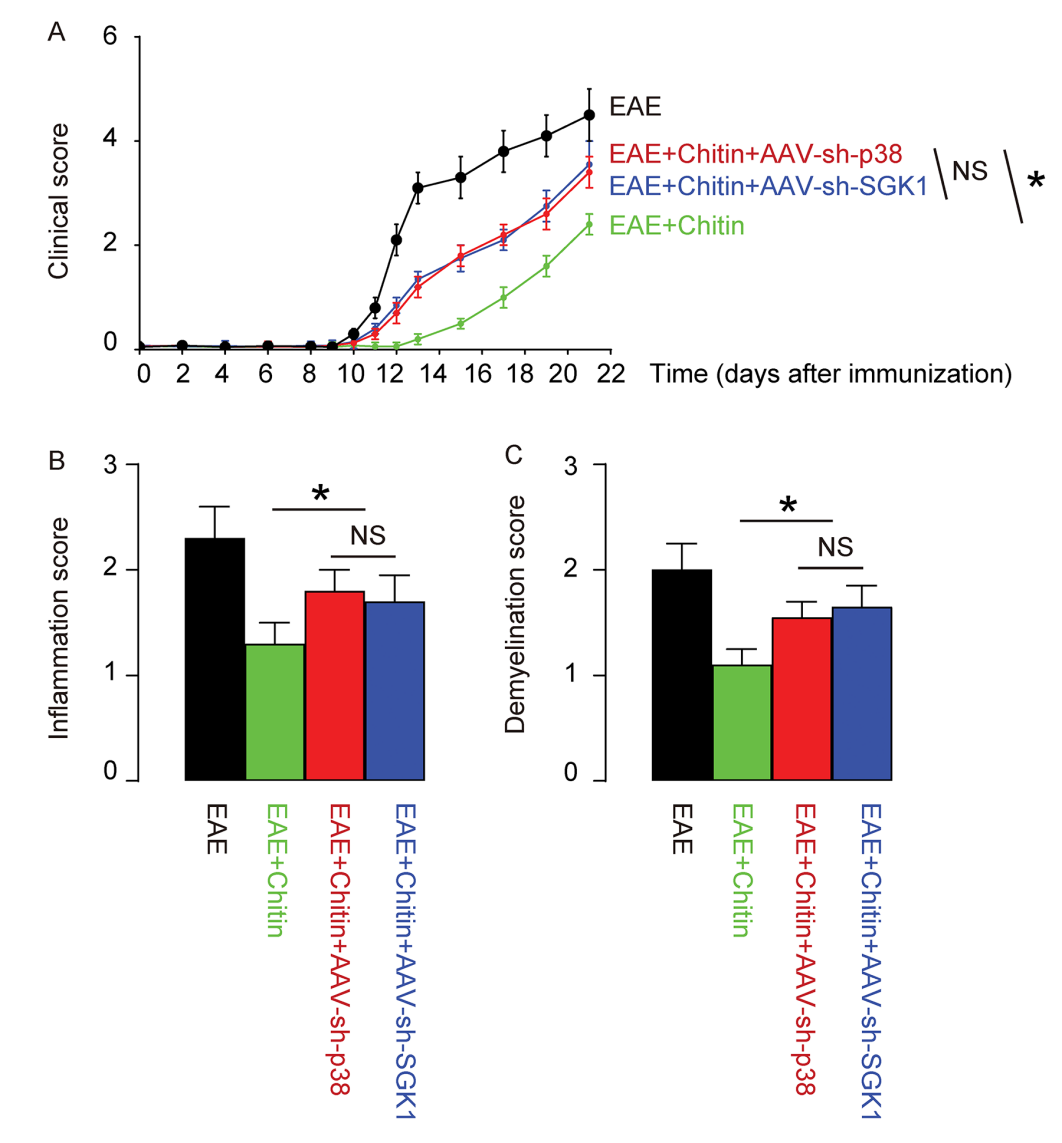
**Treatment with AAV-sh-p38 or AAV-sh-SGK1 attenuates the effects of Chitin on the severity of EAE.** MOG_35-55_ was used to immunize the mice to generate EAE model, and in vivo macrophage polarization was induced by Chitin injection. AAV-sh-p38 or AAV-sh-SGK1 was given to the mice through intraspinal injection. The development and severity of clinical signs in 4 groups of mice were monitored longitudinally till day 21 after immunization, when the mice were sacrificed to evaluate the pathological changes in the spinal cord. (**A**) The clinical score. (**B**) The inflammation score. (**C**) The demyelination score. *p<0.05. NS: non-significant. N=10.

**Figure 6:**
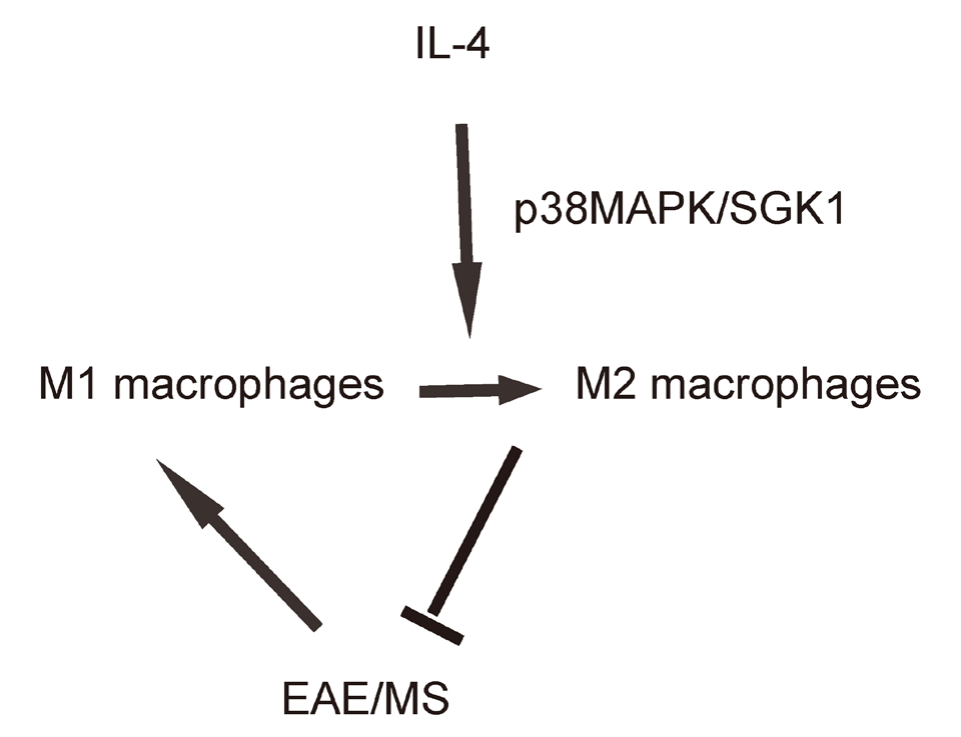
**Schematic of the study.** while M1 macrophages participate into the adverse pathogenesis in EAE, p38MAPK/SGK1 signaling induces M2 macrophage polarization, which reduces the severity of EAE.

Finally, we assessed whether p38MAPK/SGK1 signaling may be required for the M2 macrophage polarization-induced reduction in the severity of EAE. (MOG)_35-55_ was used to immunize the mice to generate EAE model, and in vivo macrophage polarization was induced by Chitin injection. AAV-sh-p38 or AAV-sh-SGK1 was given to the mice through intraspinal injection. We found that either AAV-sh-p38 or AAV-sh-SGK1 attenuated the M2 vs M1 macrophage ratio in the mouse brain from 16.6±1.8 folds’ increase to 3.5±0.5 folds’ increase and 3.7±0.4 folds’ increase, respectively, resulting in a significant attenuation on the reduction of the severity of EAE, by clinical score (Figure 5A), inflammation score (Figure 5B) and demyelination score (Figure 5C). The findings in the current study was then summarized in a schematic, showing that while M1 macrophages participate into the adverse pathogenesis in EAE, p38MAPK/SGK1 signaling induces M2 macrophage polarization, which reduces the severity of EAE (Figure 6).

## DISCUSSION

MS is a chronic autoimmune inflammatory CNS disease that affects approximately 1 million people worldwide [2]. Pathogenic T cells recognize myelin antigens in the CNS lead to focal demyelination, loss of axons, and gliosis. While CD4 T cells initiate the inflammatory cascade in the CNS, other immune and resident cells, including myeloid cells and microglia are believed to mediate oligodendrocyte damage through generation and secretion of toxic and/or proinflammatory mediators [2]. Very recently, the role of macrophages has been extensively studied in the pathogenesis of MS. Indeed, macrophages have been shown to play a substantial role in the pathogenesis of T1D [17]. Alongside the macrophages that display the classical pro-inflammatory phenotype, designated “M1” macrophages, another macrophage sub-type, designated “M2”, is responsible for wound healing and tissue-remodeling functions. The degree to which a given macrophage bears M1 or M2 characteristics is termed “polarization” [18]. While the pro-inflammatory classical macrophages (M1) enhance the progression and severity of MS through cooperation with T cells, anti-inflammatory macrophages (M2) appeared to have beneficial effects on the outcome of the disease [19,20]. M1 macrophages are characterized by high levels of reactive oxygen species (ROS), nitric oxide (NO), CD11c, TNF-α and IL-1β, while M2 macrophages are characterized by high levels of arginase 1 (Arg-1), CD206 (Mac1), CD163, CD301, Fizz1 and Ym1 [21].

Since M1 and M2 macrophages play distinct roles in MS, we hypothesized that the MS may be treated or prevented through modulation of M1/M2 transition, or polarization. Previous studies have shown that macrophage polarization may be epigenetically or genetically affected by a number of factors or signaling pathways [22]. Although Stat6 signaling is traditionally believed to the major signaling pathway that controls IL-4-induced M2 macrophage polarization, emerging evidence suggest that p38MAPK/SGK1 signaling pathway also play a critical role [23]. Indeed, here we showed the requirement of this signaling pathway in EAE-associated M2 macrophage polarization, which determines the severity of the disease [23]. EAE is characterized by neuroinflammation, demyelination, axonal damage, and progressive neurological dysfunction, which perfectly mimics the pathological changes in MS [8]. In addition, all of the currently approved therapies for MS are also efficacious in EAE, or even initially developed using EAE, which confirms the relevance of this model [24].

In the current study, suppression of p38 or SGK1 by inhibitor or shRNA showed similar effects on macrophage polarization, suggesting that SGK1 may be the most important downstream player of p38. Indeed, this signaling cascade has been well studied and shown to regulate Na+/Ca2+ Exchanger Expression and Activity in Megakaryocytes [25]. Since p38MAPK/ SGK1 may be also regulate T cell functions that contribute to the pathogenesis of EAE or MS, we need to find a way to specifically alter the levels of p38 or SGK1 in macrophages in vivo to allow determination of the specific contribution of macrophages to the disease. Thus, an adeno-associated virus (AAV) carrying sh-p38 or sh-SGK1 under the control of a CD68 promoter was used to knockdown p38 or SGK1 exclusively in macrophages [26]. CD68 is a monocyte/macrophage-specific promoter and is not activated in cells of other lineages, including T cells [26]. Use of AAVs, rather than other viral vectors is due to the less immunity caused by AAVs and their prolonged expression in the infected cells [27].

Our conclusion suggests that p38MAPK/SGK1 signaling induces M2 macrophage polarization, which reduces the severity of EAE, a model for MS. These findings imply that in vivo modulation of macrophage polarization may be a promising novel treatment for MS.

## MATERIALS AND METHODS

### Protocol approval

The study protocol and experimental design was approved by the Ethical Committee for Animal Studies at Bethune International Peace Hospital. All mouse experiments were approved by the Institutional Animal Care and Use Committee at Bethune International Peace Hospital (Animal Welfare Assurance).

### Macrophage isolation and culture

Macrophages were prepared from bone marrow, as described [28]. Macrophage were cultured in RPMI containing 10% FBS. Nonadherent cells were removed 2 h after seeding by extensive washing with medium. Cultured macrophages received 20 ng/mL of IL-4 (Sigma-Aldrich, St. Louis, MO, USA) for M2 induction. SB203580 (Sigma-Aldrich) is an inhibitor of p38, used at a concentration of 10 μM. SI113 is an inhibitor of SGK1, used at a concentration of 20 μM. Mock pretreatment was performed with vehicle control (Vh; DMSO, Sigma-Aldrich).

### Analysis of CD206 by flow cytometry

CD206+ cell analysis and sorting were performed by flow cytometry, after the cells were labeled with PEcy7-conjugated anti-CD206 antibodies (Becton-Dickinson Biosciences, San Jose, CA, USA). Flow cytometry was performed using a FACSAria (Becton-Dickinson Biosciences) flow cytometer. Negative controls were applied to remove background noise and to confirm positive cells. Data were analyzed and quantified using Flowjo software (Flowjo LLC, Ashland, OR, USA).

### Preparation of AAVs

The plasmids that contain shRNA for p38 or SGK1 were all purchased from Origene (Beijing, China). Transfection was performed with 2µg plasmids using the Lipofectamine 3000 according to the manufacturer’s instructions (Invitrogen, St. Louis, MO, USA). An adeno-associated virus (AAV) carrying sh-p38 or sh-SGK1, or a scrambled sequence under the control of a CD68 promoter was generated using the prepared sh-p38 or sh-SGK1 or a scrambled sequence to specifically target macrophages with an AAV purification kit (Clontech, Mountain View, CA, USA) as instructed.

### Mouse treatment

Male C57/BL6 mice were purchased from the SLAC Laboratory Animal Co. Ltd (Shanghai, China) were fed on standard pellet chow and water. Animals were kept at 25 °C, 50–60% humidity and a 12 h light/dark cycle with free access to water and food. Mice at 12 weeks of age were used for experiments. EAE was induced by subcutaneous injection with 250 μg MOG_35-55_ peptide (Lysine Bio-system, Xian, China) emulsified in complete Freund's adjuvant (CFA, Sigma-Aldrich) containing 4 mg/ml of heat-killed Mycobacterium tuberculosis. At 0 hour and 48 hours after immunization, mice were injected intraperitoneally with 500 ng pertussis toxin (Alexis, San Diego, CA, USA). Chitin (300 ng, Sigma-Aldrich) was injected into the spinal cord to induce recruitment and polarization of M2 cells. 10^10^ viral particles of AAV-sh-p38 or AAV-sh-SGK1 was given to the mice through intraspinal injection.

### RNA isolation and reverse transcription quantitative PCR (RT-qPCR)

Total RNA was isolated from cells with RNeasy kit (Qiagen, Hilden, Germany), and was then used as templates for cDNA synthesis with an Omniscript reverse transcription kit (Qiagen). Quantitative PCR (RT-qPCR) were performed in duplicates with QuantiTect SYBR Green PCR Kit (Qiagen). Data were collected and analyzed using 2-△△Ct method. Values of genes were first normalized against β-actin, and then compared to the experimental controls. Primer used for quantitative PCR sequences were as follows: Arg-1: TGAGAGACCACGGGGACCTG, GCACCACACTGACTCTTCCATTC; Fizz1 (Retnla: resistin-lke-α): CCATAGAGAGATTATCGTGGA, TGGTCGAGTCAACGAGTAAG; Ym1 (Chi3l3: chitinase 3-like 3): TGGAATTGGTGCCCCTACAA, AACTTGCACTGTGTATATTG; β-actin: AAATCTGGCACCACACCTTC, GGGGTGTTGAAGGTCTCAAA.

### Western blotting

Protein was extracted with RIPA lysis buffer (Sigma-Aldrich) on ice. Protein concentration was determined using a BCA protein assay kit (Bio-rad, China). Primary antibodies were rabbit anti-phosphorylated p38 (p-p38) and anti-β-actin (Cell Signaling, San Jose, CA, USA). Secondary antibody is HRP-conjugated anti-rabbit (Jackson ImmunoResearch Labs, West Grove, PA, USA). β-actin was used as a protein loading control. The protein levels were first normalized to β-actin, and then normalized to the experimental controls.

### Arginase activity measurement

Arginase activity was assessed in cell lysates indirectly by measuring urea concentration generated by the arginase-dependent hydrolysis of l-arginine. Briefly, cells were lysed with RIPA buffer (Sigma-Aldrich) for 30 min at room temperature. Standards were prepared by serially diluting a stock of urea (Sigma-Aldrich) to yield a standard range from 25 to 1500 μg/mL. Lysates and standards (25 μL) were mixed with 25 μL of 10 mM MnCl2 in 50 mM Tris-HCl (pH 7.5) in a 2 mL Eppendorf tube. Tubes were then incubated for 10 min at 55°C for activation. Next, arginine hydrolysis was conducted by incubating 50 μL of the lysates and standards with 50 μL of 0.5 M l-arginine at 37°C for 75 min, followed by the addition of 400 μL stopping solution. To measure the amount of urea in each tube, 50 μL of 9% 1-phenyl-1,2-propanedione-2-oxime (Sigma-Aldrich) in 100% ethanol was added to each sample and standard, and tubes were incubated at 100°C for 60 min. Tubes were placed in the dark at 25°C for 30 min. Samples and standards (100 μL/well) were transferred in triplicate to a 96-well plate, and optical density was read at 540 nm with a 690 nm correction. Sample concentrations were determined from the standard curve and converted to Arginase units.

### Clinical score

EAE was scored according to a 0–5 scale as follows (15): 1, limp tail or waddling gait with tail tonicity; 2, waddling gait with limp tail (ataxia); 2.5, ataxia with partial limb paralysis; 3, full paralysis of one limb; 3.5, full paralysis of one limb with partial paralysis of second limb; 4, full paralysis of two limbs; 4.5, moribund; and 5, death.

### Pathological scores

On day 21 post immunization, mouse spinal cords were harvested. H&E or Luxol fast blue (myelin stain) was done. Slides were assessed in a blinded fashion for inflammation and demyelination. For inflammation, the following scale was used: 0, none; 1, a few inflammatory cells; 2, organization of perivascular infiltrates; and 3, increasing severity of perivascular cuffing with extension into the adjacent tissue. For demyelination, the following scale was used: 0, none; 1, rare foci; 2, a few areas of demyelination; and 3, large (confluent) areas of demyelination.

### Statistical analysis

All of the statistical analyses were performed using the GraphPad Prism 6 (GraphPad Software, San Diego, CA, USA). Statistical analysis of group differences was carried out using a two-way analysis of variance (ANOVA) test followed by followed by Turkey multiple comparison post-hoc analysis. All values represent the mean ± standard deviation (SD). A value of p<0.05 was considered statistically significant after Bonferroni correction.
